# Temporal trends and black–white disparity in mortality among hospitalized persons living with HIV in the United States

**DOI:** 10.1097/MD.0000000000014584

**Published:** 2019-03-01

**Authors:** Hamisu M. Salihu, Chelsea Henshaw, Jason L. Salemi, Deepa Dongarwar, Usman J. Wudil, Omonike Olaleye, Nupur Godbole, Anjali Aggarwal, Muktar H. Aliyu

**Affiliations:** a Center of Excellence in Health Equity, Training and Research; b Department of Family & Community Medicine, Baylor College of Medicine, Houston, TX; c Vanderbilt Institute for Global Health, Vanderbilt University Medical Center, Nashville, TN; d College of Pharmacy and Health Sciences, Texas Southern University, Houston, TX; e Department of Health Policy & Vanderbilt Institute for Global Health, Vanderbilt University Medical Center, Nashville, TN.

**Keywords:** antiretroviral therapy, HIV/AIDS, in-hospital mortality, racial disparity, trends

## Abstract

We sought to determine whether black–white gap in mortality exists among hospitalized HIV-positive patients in the United States (US). We hypothesized that in-hospital mortality (IHM) would be similar between black and white HIV-positive patients due to the nationwide availability of HIV services.

Our analysis was restricted to hospitalized HIV-positive patients (15–49 years). We used the National Inpatient Sample (NIS) that covered the period from January 1, 2002 to December 31, 2014. We employed joinpoint regression to construct temporal trends in IHM overall and within subgroups over the study period. We applied multivariable survey logistic regression to generate adjusted odds ratios (OR) and 95% confidence intervals (CI).

The total number of HIV-related hospitalizations and IHM decreased over time, with 6914 (3.9%) HIV-related in-hospital deaths in 2002 versus 2070 HIV-related in-hospital deaths (1.9%) in 2014, (relative reduction: 51.2%). HIV-related IHM among blacks declined at a slightly faster rate than in the general population (by 56.8%, from 4.4% to 1.9%). Among whites, the drop was similar to that of the general population (51.2%, from 3.9% to 1.9%). Although IHM rates did not differ between blacks and whites, being black with HIV was independently associated with a 17% elevated odds for IHM (OR = 1.17; 95% CI = 1.11–1.25).

In-hospital HIV-related deaths continue to decline among both blacks and whites in the US. Among hospitalized HIV-positive patients black–white disparity still persists, but to a lesser extent than in the general HIV population. Improved access to HIV care is a key to eliminating black–white disparity in HIV-related mortality.

## 1 Introduction

With the advent of combination antiretroviral therapy (ART) in the 1990s, a notable decline in opportunistic infections led to improved survival of HIV-positive individuals.^[1–3]^ The arrival of highly effective ART regimens resulted in a significant increase in the quantity and quality of life of HIV-positive patients.^[3–5]^ The number of deaths from HIV-related complications decreased from an estimated 1.9 million in 2005 to 1.0 million in 2016 worldwide.^[6]^ These advances are attributed to a global effort crowned by the increased availability of ART and the accompanying decrease in HIV transmission rates.^[7]^ As of 2017, globally more than 20 million HIV-infected people were being managed with ART, which represents an astounding improvement from the 7.7 million people on ART in 2010.^[6]^

In the United States (US), there were an estimated 38,500 new HIV infections in 2015, which represented a decline of 8% from 2010 numbers (41,800).^[8]^ Although blacks comprise only 12% of the general US population, they account for almost half of all new HIV diagnoses, making them the most HIV-affected racial group in the US. These trends are also notable in HIV mortality, with blacks accounting for 52% of HIV-related deaths in 2015.^[9]^ Previous studies have reported a widening black–white disparity in mortality within the general population,^[10]^ and a recurring influential factor is access to care.^[11]^ We, therefore, sought to determine whether the widening black–white gap was observable among hospitalized HIV patients with access to care. We hypothesized that in-hospital mortality (IHM) would be similar between black and white HIV-positive patients as a result of availability of care. To reduce the impact of co-morbidity that tends to rise and be disproportionate with age, we restricted our analysis to hospitalized HIV-infected patients within the age bracket of 15 to 49 years.

## 2 Materials and methods

We analyzed data that contained publically-available information on US hospital discharges from January 1, 2002 to December 31, 2014 using the National Inpatient Sample (NIS). The compilation of the NIS database was implemented by the Healthcare and Cost Utilization Project (HCUP) which makes it available to researchers on an annual basis. The data was collected from participating nonfederal community hospitals throughout the country stratified by 5 characteristics: rural/urban location, number of beds, geographic region, teaching status, and ownership. Using a systematic sampling technique, 20% of the hospitals from each stratum were randomly selected to ensure geographic representativeness.^[12]^ As from the year 2012, this technique was modified to permit sample gathering from all participating hospitals. After a hospital was sampled, all inpatient hospitalization records for the facility were then captured in the NIS database. Further, HCUP provides discharge-level sampling weights so that national frequency and prevalence estimates will account for the 2-stage cluster sampling design. The NIS contains approximately 7 million inpatient hospitalizations each year (equivalent to 36 million when weighted) and the number has progressively grown as other states join the consortium.

For the purpose of this study, we restricted the study population to hospitalized individuals (15–49 years) with a positive diagnosis of HIV. To identify these patients, we scanned International Classification of Diseases, Ninth Revision, Clinical Modification (ICD-9-CM) codes (the principal diagnosis and up to 24 secondary diagnoses) in each patient's discharge record for an indication of any one of the following ICD-9 codes:

a) 042: human immunodeficiency virus (HIV) disease;
b) 079.53: HIV, type 2 (HIV-2); and
c) V08: asymptomatic HIV infection status.

Individual-level socio-demographic factors reported in the NIS database were extracted and included in the analysis. Age in years was classified into 3 categories: 15 to 24, 25 to 34, and 35 to 49. Self-reported race and ethnicity data were collected under the classification of Non-Hispanic white, Non-Hispanic black, Hispanic, or other, but we limited the dataset to only blacks and whites non-Hispanics. Median household income was classified into quartiles based on the patient's zip code, serving as a proxy for socioeconomic status. Primary insurance payers were grouped into 3 categories: government (Medicare/Medicaid), private (commercial carriers, health maintenance organizations, and preferred provider organizations), and other (self-pay, charity, etc). Hospital characteristics were also considered, including US census region (Northeast, Midwest, South, or West) and hospital type (rural, urban teaching, or urban non-teaching). In-hospital death was the main outcome considered in the study. In-hospital death was defined as a documented disposition of “expired”, indicating death before hospital discharge.

### 2.1 Statistical analysis

We employed joinpoint regression to construct the temporal trends in IHM for the period of the study overall, as well as within subgroups. Joinpoint regression is of utility in defining key periods in time that characterize changes in the rate of events over time.^[13,14]^ The iterative model-building process was initiated by fitting the yearly rate data to a straight line with no joinpoints, which assumed a single trend that best described the data points. A joinpoint—reflecting a change in the trend—was then added to the model. A Monte Carlo permutation test assessed the improvement in model fit. The process continued until a final model with an optimal (best-fitting) number of joinpoints was selected, with each joinpoint indicating a change in the trend. An annual percent change was estimated to characterize how the rate changed within each distinct trend segment.

We calculated descriptive statistics, including rates, percentages, and ratios to analyze the relationship between patient characteristics and mortality outcomes. We employed multivariable survey logistic regression to generate adjusted odds ratios (OR) that quantified the magnitude of the association between exposure status (race) and mortality among HIV-positive patients. Statistical analyses were performed with SAS, version 9.4 (SAS Institute, Inc., Cary, NC); we assumed a 5% type I error rate for all hypothesis tests (2-sided). This study was approved as exempt by the Baylor College of Medicine Institutional Review Board.

## 3 Results

In total, 56,203 (2.9%) cases of IHM due to HIV were reported over the study period (2002 through 2014). The total number of HIV-related hospitalizations and HIV-related in-hospital deaths decreased progressively over time, with 6914 (3.9%) HIV-related in-hospital deaths in 2002 versus 2070 HIV-related in-hospital deaths (1.9%) in 2014. The proportion of HIV-positive men (3.1%) who died was higher than that of HIV-positive women (2.5%) during the entire period, with slightly more non-Hispanic blacks (2.9%) dying than non-Hispanic whites (2.7%). Similarly, more deaths occurred among non-Hispanic black men (3.2%) and women (2.6%) than in non-Hispanic white men (2.9%) and women (2.3%). The overall IHM was similar between black and white women (2.6% vs 2.3%, respectively *P* >.05.

**Figure 1:**
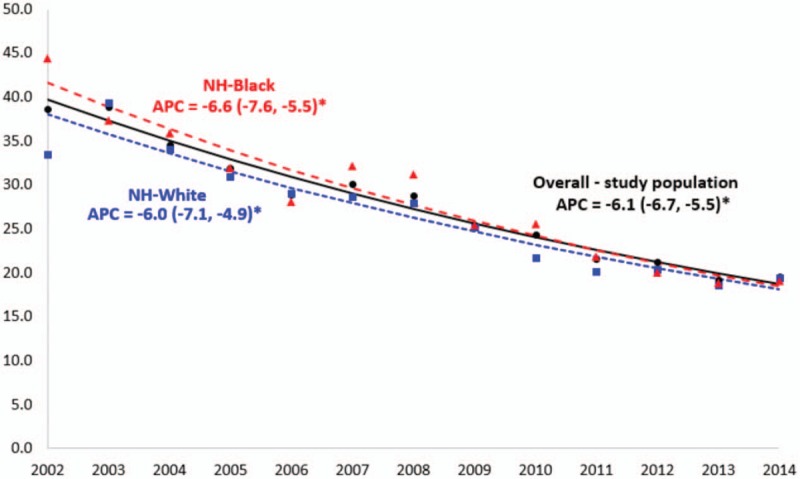
Temporal trends in the prevalence of in-hospital mortality (per 1000 hospitalizations) among HIV-positive men and women, overall and by race/ethnicity, United States, 2002 to 2014. APC = annual percent change; NH = non-Hispanic.

Figure 1 compares temporal trends in IHM among HIV-positive patients in the US for whites versus blacks. In the entire HIV-positive population as well as within the 2 racial categories, there was a significant reduction in IHM. The IHM rate dropped significantly over the study period, from 3.9% to 1.9%, equivalent to a relative reduction of 51.2%. There was a 56.8% decrease in IHM among blacks (from 4.4% to 1.9%), while among whites, the drop was 51.2% (from 3.9% to 1.9%), similar to that of the entire population. There was no statistically significant difference in annual percent change in IHM between the 2 racial groups.

**Figure 2:**
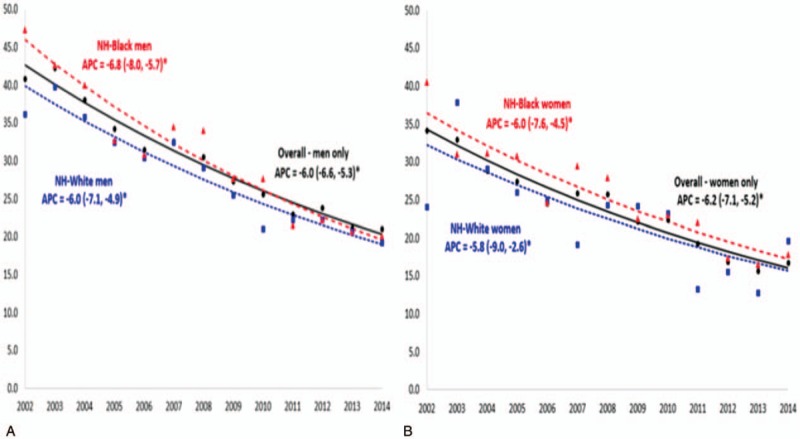
A, B. Temporal trends in the prevalence of in-hospital mortality (per 1000 hospitalizations) among HIV-positive men and HIV-positive women, overall and by race/ethnicity, United States, 2002 to 2014. APC = annual percent change; NH = non-Hispanic.

We further explored racial disparity by sex separately with respect to temporal trends. The results are shown in Figure 2a (for men) and Figure 2b (for women). Among men, the IHM rate for whites dropped from 3.6% to 1.9% by the end of the study period, equivalent to a 47.2% decline. The greatest decline was among black men and women (by 57.4% and 56.1%, respectively), while the lowest decline was noted among white women, who experienced a drop of only 16.7% in IHM. Although both black men and women had markedly elevated levels of IHM at the beginning of the study period (4.7% and 4.1% compared to 3.6% and 2.4% for whites respectively), mortality for black women was the lowest by the end of the study (1.8%). The reduction in IHM did not demonstrate statistically significant black–white differences when stratified by sex.

Table 1 summarizes the predictors of IHM among HIV-positive individuals in the US. Age of the patient was predictive of mortality, with older individuals having between 50% to 90% greater odds of mortality in a dose-response pattern (i.e., increasing odds with age). HIV-positive women had a 18% lower likelihood of death compared to HIV-positive men (adjusted OR, aOR = 0.82, 95% confidence interval [CI] = 0.78 - 0.85), while black patients had a 17% greater odds of death than their white counterparts (aOR = 1.17, 95% CI = 1.11–1.25). Patients covered by public health insurance exhibited lower odds of mortality compared to those on private health insurance (aOR = 0.88, 95% CI = 0.83–0.95. Individuals living in the Western and Southern US also demonstrated greater odds for death than those living in the Northeast. There was no association between IHM on the one hand, and household income, hospital size, and urban/rural hospital location.

**Table 1:**
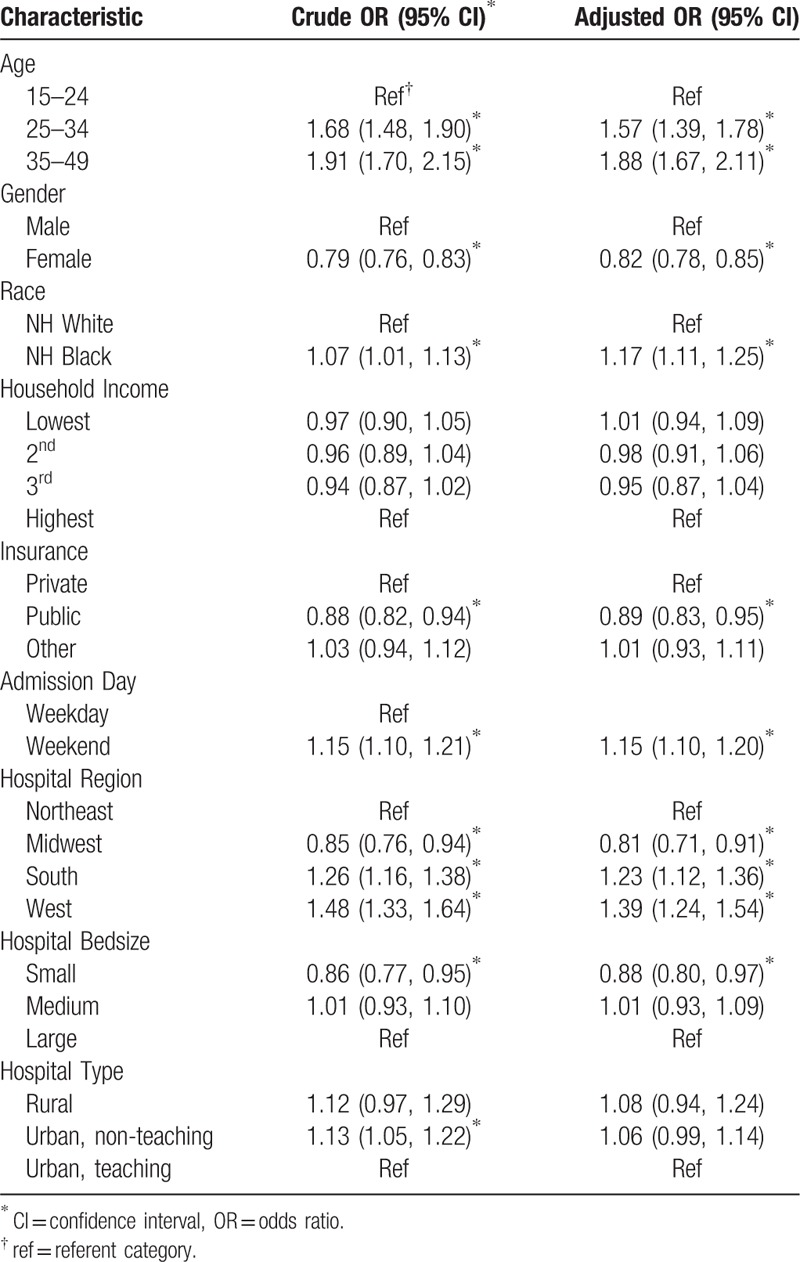
Predictors of in-hospital mortality among hospitalized HIV-positive patients in the United States, 2002 to 2014.

In Table 2, we examined the same predictors stratified by race/ethnicity, that is, separate analysis for non-Hispanic blacks and non-Hispanic whites. Similar results were obtained for both racial groups as for the entire group with respect to age, sex, household income, and urbanicity. The elevated odds of IHM for HIV-positive patients residing in the Southern US was particularly remarkable among blacks, while their white counterparts did not experience significant risk elevation.

**Table 2:**
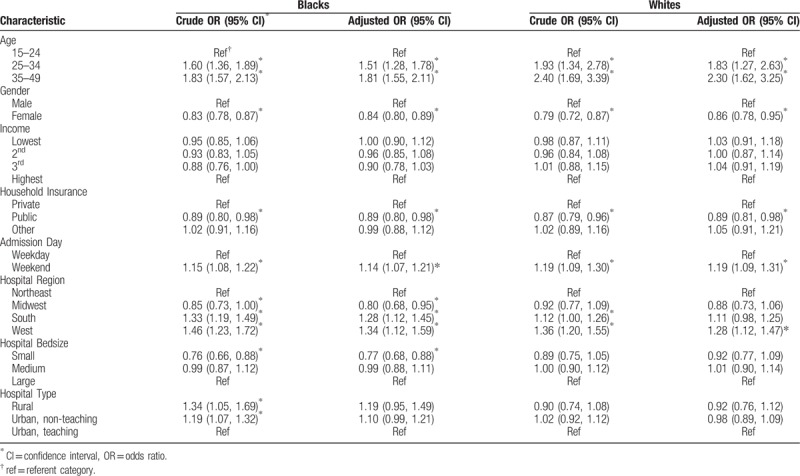
Predictors of in-hospital mortality among HIV-positive blacks and whites (men and women), 2002 to 2014.

Since sex could play an important role either as a confounder or a mediator for the black–white disparity in mortality, we generated the same results for men and for women separately. Among HIV-positive men (Table 3), black-white differences in predictors of mortality were observed in regards to health insurance, weekend admission, and hospital size. Whereas public health insurance was protective of mortality, weekend admission was a risk factor for mortality among white men only. Among black men only, small hospital size significantly reduced the odds of mortality (aOR = 0.79, 95% CI = 0.69–0.92). Public insurance coverage, residence in the southern US, small hospital bed size and urban hospital location were significant predictors of IHM among HIV-positive black women, but not their white counterparts (Table 3). Weekend admission was predictive of mortality in both black and white women.

**Table 3:**
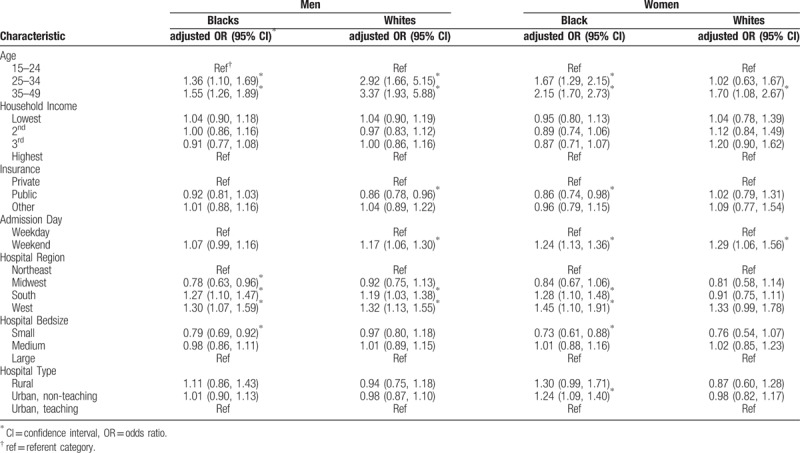
Predictors of in-hospital mortality among HIV-positive blacks and whites (men and women), 2002 to 2014.

## 4 Discussion

In this large nationwide study on racial disparity in mortality among hospitalized HIV-positive young individuals, we found a 51.2% reduction in IHM over the period 2002 to 2014. The magnitude of the reduction in IHM over the study period was similar for whites and for blacks (51.2% vs 56.8%, respectively). However, despite this significant and appreciable reduction in IHM in both racial groups, adjustment for potential confounders revealed that HIV-positive blacks were 17% more likely to experience death following hospital admission when compared to their white counterparts. Previous studies based on national vital records reported widening of HIV-related black-white mortality gap when the mortality rates for the period 1990 to 1994 were compared to those for the period 2005 to 2009.^[10]^ One major difference between that study and ours is that we restricted our analysis to IHM. Secondly, we examined mortality among a relatively younger population (15–49 years) with greater life expectancy than for the general population, and within which black young adults exhibit more than twice the mortality risk of their white counterparts.^[15–18]^ Our findings could, therefore, be an indication that the level of HIV-related black-white gap in mortality among hospitalized patients exposed to supervised management within hospital institutions was lower than that of the general population of HIV patients. Access to care is, therefore, a major factor that could explain previously reported widening black-white disparity in the HIV general population. The availability of care within the hospital setting provides an avenue for HIV-related health challenges to be properly addressed, including diagnosis of HIV infection, linkage to care, retention in care, receipt of antiretroviral therapy, achievement of viral suppression and management of HIV co-morbidities, such as tuberculosis.^[19,20]^ Since whites attain viral suppression better than blacks,^[21]^ HIV-related IHM would be expectedly lower among whites, as observed in this study. However, our analysis in this regard was weakened by the lack of information on HIV viral load as well as other biologic predictors of survival.

The black-white disparity in IHM within this study could be explained by non-biologic factors related to quality of care upon hospitalization, pre-hospitalization anti-retroviral therapy (ART) uptake and adherence since these parameters influence HIV morbidity and mortality. HIV-positive black people are less likely to adhere to medical appointments and to achieve HIV suppression.^[22,23]^ The decreased likelihood of optimal HIV disease control before hospitalization and the elevated risk for disease progression may be a plausible explanation for the 17% increment in mortality after adjustment for other factors. It is also reasonable to suggest that inequity in accessing quality care by HIV-positive individuals even during admission could be a contributory factor.

As in previous studies,^[24]^ we found disparity in mortality in relation to place of residence. We observed that living in the southern US was associated with elevated risk of mortality during hospitalization among black, but not white HIV-positive patients. Previous studies did report that HIV-infected people residing in the US South experienced worse outcomes in general than those living in other geographical regions of the US.^[25]^ Research has shown that the US South bears the highest age-standardized HIV mortality rates and the highest age-standardized HIV case fatality rates.^[26]^ This geographical disparity may be explained by the preponderance of HIV infections in the US South, as well as adverse health outcomes among HIV-positive individuals intimately linked to unfavorable institutional policies in many southern states, including refusal to broaden opportunities for low-income earners to have access to healthcare (e.g., through Medicaid expansion) and the high rates of incarceration in these states that disproportionately affect blacks.^[11]^ These factors represent a huge barrier to achieving virologic suppression among HIV-positive US black southerners, making them sicker and more likely to die during hospitalization.^[27]^

### 4.1 Limitations

A limitation in the data we analyzed is the absence of information regarding quality of HIV care or stratification of outcomes by treatment cascade, which is an outline of the essential steps to viral suppression and therefore, reduction of mortality risk. These steps encompass diagnosis of HIV (and whether delayed or not), linkage to care, retention in care, receipt of ART, and achievement of viral suppression.^[28]^ Other important data that could have impacted our analysis but were absent in the dataset included HIV disease severity, types of treatment received, level of HIV viral load during the phase of hospital admission and social support systems. Nonetheless, this study has merits, including a large study population sample, gathered nationwide, making our findings generalizable. To our knowledge, this is the first study that examined black-white disparity in IHM among HIV-positive patients in the US, and the results represent new findings that could be of utility to policy makers and HIV care providers in general.

### 4.2 Future directions

Future research should include considerations of patient-level data that could impact survival, such as severity or clinical stage of HIV illness. Future exploration of potential mechanisms by which societal stigma and discrimination might moderate racial/ethnic disparities in HIV-related mortality will also be appropriate.

## 5 Conclusion

In summary, we found a declining trend in rates of HIV-associated IHM between 2002 and 2014 among a large, population-based sample of HIV-positive persons in the US. IHM was associated with age, sex, and geographical region. There was also a consistent decline in Black-white disparity in in-hospital death over the period of the study. We recommend continued access to high-quality HIV treatment and prevention services for all racial/ethnic groups in the US.

## Acknowledgments

We thank the HCUP for providing access to the NIS database.

## Author contributions

**Conceptualization:** Hamisu M. Salihu, Chelsea Henshaw, Jason L. Salemi, Omonike Olaleye, Nupur Godbole, Anjali Aggarwal, Muktar Hassan Aliyu.

**Data curation:** Deepa Dongarwar, Omonike Olaleye, Nupur Godbole.

**Formal analysis:** Hamisu M. Salihu, Chelsea Henshaw, Jason L. Salemi, Deepa Dongarwar, Usman J. Wudil, Omonike Olaleye, Nupur Godbole, Anjali Aggarwal, Muktar Hassan Aliyu.

**Investigation:** Chelsea Henshaw, Jason L. Salemi, Omonike Olaleye, Muktar Hassan Aliyu.

**Methodology:** Hamisu M. Salihu, Chelsea Henshaw, Jason L. Salemi, Deepa Dongarwar, Usman J. Wudil, Nupur Godbole, Anjali Aggarwal, Muktar Hassan Aliyu.

**Project administration:** Usman J. Wudil.

**Supervision:** Jason L. Salemi.

**Validation:** Hamisu M. Salihu, Chelsea Henshaw, Jason L. Salemi, Deepa Dongarwar, Usman J. Wudil, Anjali Aggarwal, Muktar Hassan Aliyu.

**Writing - original draft:** Hamisu M. Salihu, Chelsea Henshaw, Jason L. Salemi, Deepa Dongarwar, Usman J. Wudil, Nupur Godbole, Anjali Aggarwal, Muktar Hassan Aliyu.

**Writing - review & editing:** Hamisu M. Salihu, Chelsea Henshaw, Jason L. Salemi, Deepa Dongarwar, Usman J. Wudil, Nupur Godbole, Anjali Aggarwal, Muktar Hassan Aliyu.
